# Insulin-mediated muscle microvascular perfusion and its phenotypic predictors in humans

**DOI:** 10.1038/s41598-021-90935-8

**Published:** 2021-06-01

**Authors:** Kaitlin M. Love, Linda A. Jahn, Lee M. Hartline, James T. Patrie, Eugene J. Barrett, Zhenqi Liu

**Affiliations:** 1 Division of Endocrinology and Metabolism, Department of Medicine, University of Virginia Health System, Charlottesville, VA USA; 2 Department of Public Health Sciences, University of Virginia Health System, Charlottesville, VA USA

**Keywords:** Physiology, Endocrinology, Medical research

## Abstract

Insulin increases muscle microvascular perfusion and enhances tissue insulin and nutrient delivery. Our aim was to determine phenotypic traits that foretell human muscle microvascular insulin responses. Hyperinsulinemic euglycemic clamps were performed in 97 adult humans who were lean and healthy, had class 1 obesity without comorbidities, or controlled type 1 diabetes without complications. Insulin-mediated whole-body glucose disposal rates (M-value) and insulin-induced changes in muscle microvascular blood volume (ΔMBV) were determined. Univariate and multivariate analyses were conducted to examine bivariate and multivariate relationships between outcomes, ΔMBV and M-value, and predictor variables, body mass index (BMI), total body weight (WT), percent body fat (BF), lean body mass, blood pressure, maximum consumption of oxygen (VO_2_max), plasma LDL (LDL-C) and HDL cholesterol, triglycerides (TG), and fasting insulin (INS) levels. Among all factors, only M-value (r = 0.23, p = 0.02) and VO_2_max (r = 0.20, p = 0.047) correlated with ΔMBV. Conversely, INS (r = − 0.48, p ≤ 0.0001), BF (r = − 0.54, p ≤ 0.001), VO_2_max (r = 0.5, p ≤ 0.001), BMI (r = − 0.40, p < 0.001), WT (r = − 0.33, p = 0.001), LDL-C (r = − 0.26, p = 0.009), TG (r = − 0.25, p = 0.012) correlated with M-value. While both ΔMBV (p = 0.045) and TG (p = 0.03) provided significant predictive information about M-value in the multivariate regression model, only M-value was uniquely predictive of ΔMBV (p = 0.045). Thus, both M-value and VO_2_max correlated with ΔMBV but only M-value provided unique predictive information about ΔMBV. This suggests that metabolic and microvascular insulin responses are important predictors of one another, but most metabolic insulin resistance predictors do not predict microvascular insulin responses.

## Introduction

Universally acknowledged as fundamental in the pathogenesis of type 2 diabetes (T2D), metabolic insulin resistance, defined as diminished insulin-mediated glucose utilization, is also reliably found in people with obesity and type 1 diabetes (T1D)^1,2^. While comprehensive mechanisms for insulin resistance in obesity and T2D have been extensively studied, those in T1D remain to be fully elucidated^3^. Insulin resistance is clearly detrimental. Even in individuals without diabetes, reduced insulin-mediated glucose utilization correlates with increased coronary artery disease^4^. In T1D, insulin resistance correlates with increased microvascular and macrovascular complications including cardiovascular disease^5^. Phenotypic factors associated with metabolic insulin resistance have been well delineated over the past several decades, using the euglycemic hyperinsulinemic clamp technique as the gold standard measure as well as other estimates such as homeostasis model assessment-insulin resistance (HOMA-IR), quantitative insulin sensitivity check index (QUICKI), and McAuley index^6–8^.

Insulin resistance can develop at all insulin target tissues including skeletal muscle^9^, cardiac muscle^10^, adipose tissue^11^, liver^12^, as well as the vasculature^13^. Insulin’s actions at the microvasculature play a particularly important role in euglycemia and tissue health because capillaries are the site of nutrient and waste exchange^13^. Mounting evidence suggests that insulin action in the muscle microvasculature closely couples with its action on the skeletal myocytes. In health, insulin vasodilates muscle microvasculature to increase its perfusion and facilitate its own trans-endothelial transport to the muscle interstitium^13^. Insulin’s effect on muscle microvascular perfusion occurs within 15–30 min, and precedes insulin-mediated muscle glucose uptake^14^. Inhibition of insulin’s microvascular action decreases insulin-mediated muscle glucose disposal by up to 40%^14^. Additionally, diseases like T2D and obesity that are characterized by metabolic insulin resistance, often manifest with diminished or absent vasodilatory responses to insulin^2,15^.

Microvascular insulin resistance appears early in diet-induced obesity^16^ and is present in normoglycemic humans with only class 1 obesity^2^. In healthy humans receiving lipid infusions, early microvascular responses to insulin strongly associate with steady-state skeletal muscle insulin-mediated glucose uptake, and physical fitness correlates with both metabolic and vascular insulin responsiveness^17^. Similarly, vascular insulin resistance is clearly present in T1D^15^, and, in middle-aged individuals with T1D, steady-state insulin mediated glucose uptake strongly correlates with microvascular insulin responsiveness^18^. While most data derived from animal studies confirm the co-existence of metabolic and microvascular insulin resistance^14,16,19^, whether factors that predict metabolic insulin resistance also predict microvascular responses to insulin in humans remains less clear. This is important as vascular insulin resistance contributes to the development of metabolic insulin resistance and occurs before metabolic insulin resistance; thus, identifying factors associated with developing microvascular insulin resistance, compared to metabolic insulin resistance, may provide important biomarkers to allow for earlier detection of insulin resistance and cardiovascular risks in individuals with or without obesity and/or diabetes.

In the current study, we sought to determine whether risk factors for metabolic insulin resistance would also predict microvascular insulin responses in humans with a large range of insulin action and resistance patterns.

## Methods

In order to sample from a range of insulin resistance patterns without the confounding influence of clinically significantly metabolic disarrays (e.g. hypertension, dyslipidemia), we prospectively studied 97 adult human subjects who were either lean and healthy (i.e. normal beta-cell function and minimal evidence of insulin resistance), had a diagnosis of class I obesity without other comorbidities (i.e. metabolic insulin resistance without clinically significant dysglycemia), or controlled T1D without complications or other comorbidities (i.e. insulin deficient but receiving appropriate replacement). Portions of this data set were previously reported^20–22^. The studies were performed at the University of Virginia (UVA) Clinical Research Unit (CRU) under study protocols approved by the UVA Institutional Review Board (IRB) and in accordance with the World Medical Association’s 2013 Declaration of Helsinki.

All subjects were between 18 and 46 years old. Three participants over 40 years of age were included, all with class I obesity or T1D without other comorbidities, due to desire to sample from a wide array of insulin resistance patterns. Subjects were excluded based on use of vasoactive medications and supplements (i.e. angiotensin-converting enzyme inhibitors, angiotensin II receptor blockers, β-blockers, statins, and fish oil), hyperlipidemia, smoking history within 6 months, pregnancy or lactation, anemia, intracardiac shunt, or unstable pulmonary or cardiovascular conditions. Lean, healthy participants were excluded based on BMI > 25, first degree relative with diabetes, and chronic medical conditions. Individuals with T1D were excluded based on BMI > 29 and the presence of any diabetes-related complication. Participants with class 1 obesity were excluded based on BMI < 30 or ≥ 35, personal or family history of diabetes. Participants underwent a screening visit prior to informed consent and study enrollment to verify that inclusion and exclusion criteria were satisfied. The screening visit included a physical examination performed by a physician and blood work consisting of a complete blood count, complete metabolic panel to exclude electrolyte, renal, and liver abnormalities, lipid panel, and urine β-hCG if female. Each participant gave written informed consent before study enrollment.

### Body composition and cardiorespiratory fitness testing

After study enrollment, subjects presented for an outpatient visit involving measurement of maximum consumption of oxygen (VO_2_max) by treadmill Bruce protocol and body composition using air displacement plethysmography (BOD POD, Life Management, Concorde, CA), on a separate day from vascular and metabolic testing. Because of the known influence of sex and age on aerobic capacity, we classified each participant into one of three levels of cardiorespiratory fitness (i.e. highest representing superior or excellent, middle representing good or fair, and lowest indicating poor or very poor cardiorespiratory performance) based on American College of Sports Medicine Guidelines for fitness categories^23^.

### Measurements of metabolic and microvascular insulin responses

Subjects were admitted to the CRU at 0700 h following an overnight fast beginning at 2000 h the previous night and abstaining from exercise and caffeine for 24 h. Participants with T1D treated with multiple daily injections administered final doses of basal insulin the evening prior to admission and did not administer short acting insulin the day of admission. Individuals treated with insulin pump continued their insulin pump basal rate throughout the admission. Upon admission, an antecubital venous catheter was placed on the right arm for infusions of insulin, dextrose, microbubbles and normal saline. A second venous catheter was placed distal to the antecubital vein for blood sampling. Baseline blood samples including glucose and insulin levels (for participants without T1D) were then collected. Target glucose range for participants with T1D to begin studies was set at 4.4–8.3 mmol/L (80–150 mg/dL). Eight participants with T1D had blood glucose levels above target range (> 8.3 mmol/L or > 150 mg/dL) and received a low dose insulin infusion (0.1–0.15 mU/kg/min) beginning 2-h prior to vascular and clamp studies to achieve euglycemia.

#### Hyperinsulinemic euglycemic clamp

Hyperinsulinemic euglycemic clamp is a well validated and reproducible method for assessing insulin sensitivity and is considered the gold-standard measurement of whole-body insulin resistance^24^ and the steady-state glucose infusion rates reflect glucose uptake throughout the body but predominately at the skeletal muscle, where most glucose uptake transpires^25^. It begins with a primed (2 mU/kg/min × 10 min), continuous (1 mU/kg/min × 110 min) regular insulin intravenous (IV) infusion, and plasma glucose is measured every 5 min with 20% dextrose infused at a variable rate to maintain euglycemia. This insulin infusion regimen elevates plasma insulin concentrations to high physiologic levels (200–600 pM)^22,26^, comparable to post-prandial levels^27,28^. During the clamp, plasma glucose is maintained within ~ 0.5 mmol/L (10 mg/dL) of basal levels. The steady-state M-value was taken as the average glucose infusion rate over the final 40 min of the clamp, expressed as mg/kg/min.

Contrast-enhanced ultrasound (CEU) was used to assess muscle microvascular perfusion before and at the end of the insulin infusion. Definity^®^ microbubbles (Lantheus Medical Imaging, North Billerica, MA), a lipid coated perfluorocarbon gas, were infused intravenously with the subject in left lateral decubitus position. Once the contrast concentrations reached steady state (~ 2–3 min), transverse images of the left proximal forearm, approximately 5 cm distal to the antecubital fossa, were obtained using either a SONOS 7500 or EPIQ 7 cardiovascular ultrasound system (Philips Medical Systems; Andover, MA) at a mechanic index of 1.5, as previously described^28^. Ultrasound images were analyzed using the QLAB software (Philips Medical Systems; Andover, MA) by investigator blinded to other subject characteristics including the M-value. The intensity of the contrast signal provides an index of the volume of microvasculature perfused, or microvascular blood volume (MBV). The ΔMBV was determined by the difference between post- and pre-insulin MBV divided by pre-insulin MBV and is used as an index of muscle microvascular insulin sensitivity.

### Biochemical analysis

Screening biochemical analyses, including lipid panel, were performed at the UVA Clinical Chemistry Laboratory. LDL-C, HDL-C, and TG were determined by histochemical assay. Plasma glucose levels during insulin clamp were determined using an YSI glucose analyzer (Yellow Spring Instruments). Plasma insulin levels were determined using Siemens Healthcare Diagnostics Immulite 2000 Random Access Analyser.

### Statistical analyses

#### Data summarization

Categorical descriptive data are summarized as frequencies and percentages, and continuous scaled descriptive data are summarized by the mean and standard error of mean (SEM).

#### Univariate and multivariate analyses

The objective of this study was to determine whether factors known to predict metabolic insulin resistance also predict microvascular responses to insulin in humans. To address this objective both univariate correlation analyses and multivariate regression analyses were conducted. The univariate analyses examined the bivariate relationships between the metabolic insulin resistance risk factors and M-value or ΔMBV. The set of phenotypic variables included as potential metabolic insulin resistance risk factors were: body mass index (BMI), total body weight (WT), percent body fat (BF), lean body mass, blood pressure, cardiorespiratory fitness (VO_2_max), plasma levels of LDL cholesterol (LDL-C), HDL cholesterol, triglycerides (TG), and fasting insulin (INS). Per metabolic insulin resistant risk factor, the bivariate association was quantified via the Pearson Product Moment correlation coefficient (r). With regard to hypothesis testing, the null hypothesis for each bivariate correlation analysis was that there is no bivariate correlation between the values of metabolic insulin resistant risk factor and the values of microvascular parameter. Rejection of the null hypothesis was based on a two-sided p ≤ 0.05 threshold.

The multivariate regression analyses were focused on identifying unique bivariate relationships between the metabolic insulin resistance risk factors and the metabolic or microvascular parameters (i.e. M-value or ΔMBV). For each multivariate regression analysis, the metabolic or microvascular parameter (i.e. M-value or ΔMBV) served as the multivariate regression model dependent variable and the metabolic insulin resistance risks factors and either M-value or ΔMBV served as the multivariate regression model independent variables. With one notable exception, fasting insulin, the metabolic resistance risk factors were those examined in the aforementioned univariate analyses. With regard to hypothesis testing, a set of type III ANOVA F-tests were conducted to identify those metabolic insulin resistance risk factors that provide unique microvascular parameter predictive information not explained by any of the remaining metabolic insulin resistance risk factors. Each type III ANOVA F-test tested the null hypothesis that there is no unique bivariate association between the metabolic resistance risk factor and the microvascular parameter. Significance was established at the p ≤ 0.05 significance level.

Pairwise comparisons of mean M-value and mean ΔMBV between cardiorespiratory fitness tertiles and between females and males as well as comparisons of mean fasting insulin concentrations between healthy and obese groups were conducted via the Welch version of the Student’s t-test. Comparisons between phenotypic variables for the healthy, T1D, and obese groups were conducted via Brown-Forsythe and Welch ANOVA tests. Statistical significance was established at the p ≤ 0.05 significance level.

### Ethics approval and consent to participate

The studies were performed at the University of Virginia (UVA) Clinical Research Unit (CRU) under study protocols approved by the UVA Institutional Review Board (IRB) and in accordance with the World Medical Association’s 2013 Declaration of Helsinki. All study participants provided written informed consent to participate in the study.

### Consent for publication

All authors consent this manuscript for publication.

### Prior presentation

Portions of this study were presented in poster form at the 80th Scientific Sessions of the American Diabetes Association, Virtual Conference, June 12–16, 2020.

## Results

Data from all 97 subjects were analyzed and their characteristics were summarized in Table 1. The study cohort included 57 lean and healthy subjects (58.8%), 15 subjects with class 1 obesity (15.5%; BMI 33.7 ± 1.2 kg/m^2^), and 25 persons with T1D on insulin replacement who were otherwise healthy (25.8%). Forty-six of the 97 subjects were female (47.2%). All participants were young (age 25.4 ± 0.7; range 18–46 years), normotensive and without significant dyslipidemia. Average BMI was 24.8 ± 0.5 kg/m^2^. All subjects with T1D had controlled glycemia (HbA1c 7.4 ± 0.2%; 57 mmol/mol) with no evidence of microvascular complications. As anticipated, most phenotypic variables were different across groups with the exception of blood pressure. While the mean ΔMBV was not significantly different, the groups with T1D and obesity showed a trend towards reduction from the levels observed in the healthy participants.

**Table 1:** Participants’ characteristics (mean ± SEM).

	Combined	Healthy	T1D	Obese	P-value
Participants (n)	97	57	25	15	
Female sex (n/%)	46/47.4%	27/47.3%	12/47.6%	11/73%	
Ethnicity	Caucasian 86.7% (n = 86) African American 9.3% (n = 9) Hispanic 2% (n = 2) Asian 2% (n = 2)	Caucasian 88% (n = 50) African American 9% (n = 5) Hispanic 3.5% (n = 2) Asian 3.5% (n = 2)	Caucasian 96% (n = 24) African American 4% (n = 1)	Caucasian 80% (n = 12) African American 20% (n = 3)	
Age (years)	25.4 ± 0.7	22.9 ± 0.4	30.5 ± 1.7	26.5 ± 2.2	
Weight (kg)	76.2 ± 1.6	68.5 ± 1.2	80.8 ± 2.7	97.9 ± 4.2	< 0.001
VO_2_max (mL/kg/min)	41.5 ± 1.0	43.5 ± 1.7	42.6 ± 2.2	32.7 ± 1.7	< 0.001
Lean body mass (kg)	55.8 ± 1.2	54 ± 1.6	60.2 ± 2.4	55.9 ± 2.7	< 0.001
Body fat (%)	24.8 ± 1.1	20.8 ± 1	24.8 ± 2	39.6 ± 1.7	< 0.001
Systolic blood pressure (mmHg)	120.9 ± 1.4	119.6 ± 1.6	125.6 ± 3.2	118.4 ± 3.3	0.15
Diastolic blood pressure (mmHg)	70.0 ± 0.9	69.5 ± 1	70.9 ± 1.8	71.5 ± 2.8	0.63
LDL-C (mmol/L)	2.44 ± 0.08	2.26 ± 0.08	2.68 ± 0.17	2.76 ± 0.17	0.02
HDL-C (mmol/L)	1.48 ± 0.05	1.62 ± 0.08	1.36 ± 0.09	1.2 ± 0.09	< 0.001
Triglycerides (mmol/L)	0.81 ± 0.04	0.78 ± 0.05	0.66 ± 0.04	1.13 ± 0.19	0.04
Fasting insulin (mU/L)	4.1 ± 0.4	3.1 ± 0.2		8.1 ± 1.1	0.001*
M-value (mg/kg/min)	5.8 ± 0.2	6.3 ± 0.3	5.5 ± 0.4	4.0 ± 0.4	< 0.001
Baseline MBV (VI)	4.1 ± 0.3	4.8 ± 0.4	1.6 ± 0.1	6.1 ± 0.7	< 0.001
Post-insulin MBV (VI)	4.6 ± 0.3	5.5 ± 0.4	1.7 ± 0.2	5.8 ± 0.6	< 0.001
ΔMBV	0.18 ± 0.05	0.23 ± 0.1	0.12 ± 0.1	0.05 ± 0.1	0.32

T1D, type 1 diabetes; SEM, standard error of mean; LDL-C, low density lipoprotein cholesterol; HDL-C, high density lipoprotein cholesterol; VO_2_max, maximum oxygen consumption; ΔMBV, changes in microvascular blood volume following insulin clamp; VI, video intensity; P-values were determined by Brown-Forsythe and Welch ANOVA tests comparing the three groups. (healthy, T1D, obese).

*Indicates unpaired t-test with Welch’s correction comparing participants who are healthy and obese.

**Figure 1:**
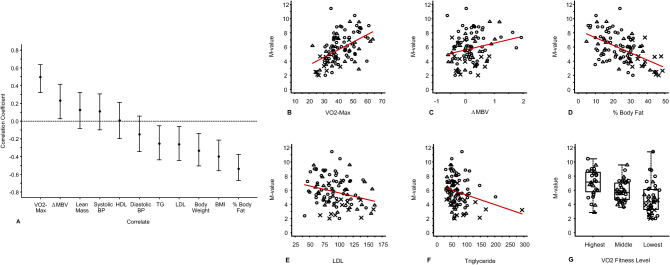
Bivariate relationships between M-value and prediction variables. (**A**) Pearson correlations between M-value and subject characteristics. The vertical lines indicate the 95% confidence interval for the correlation coefficient. (**B**–**F**) Relationships between M-value and individual predictor variables. The red lines represent the ordinary least squares linear regressions. (**G**) Relationship between M-value and cardiorespiratory fitness. ΔMBV, insulin-mediated change in muscle microvascular blood volume; BMI, body mass index; BP, blood pressure; HDL, high-density lipoprotein cholesterol; LDL, low-density lipoprotein cholesterol; TRI, triglycerides; VO_2_max, maximum consumption of oxygen (mL/kg/min); VO2 fitness, level of cardiorespiratory fitness by tertile. Plotting symbols: Healthy°, Obese˟, Type 1 diabetes^Δ^.

To determine phenotypic variables associated with metabolic insulin resistance in our study population, univariate bivariate correlation analyses were conducted between M-value and known metabolic insulin response predictors as well as ΔMBV. As shown in Fig. 1, VO_2_max (r = 0.5, 95% CI [0.32, 0.64] p < 0.001) and ΔMBV (r = 0.23, 95% CI [0.03, 0.42], p = 0.03) correlated positively with M-value. Conversely, percent body fat (r = − 0.54, 95% CI [− 0.67, − 0.38], p < 0.001), BMI (r = − 0.40, 95% CI [− 0.56, − 0.21], p < 0.001), total body weight (r = − 0.33, 95% CI [− 0.50, − 0.14], p = 0.001), LDL cholesterol (r = − 0.26, 95% CI [− 0.44, − 0.06], p = 0.009), and triglycerides (r = − 0.25, 95% CI [− 0.44, − 0.05], p = 0.012) were inversely correlated with M-value. Since fasting plasma insulin levels independently predict insulin-mediated whole body glucose disposal^7^, a subgroup analysis of bivariate relationship between M-value and fasting plasma insulin levels in the all subjects without diabetes was performed. As expected, fasting plasma insulin level was inversely associated with M-value (r = − 0.48, 95% CI [− 0.64, − 0.28], p < 0.001). Furthermore, given the moderate correlation between VO_2_max and M-value, the cohort was divided into 3 tertiles of cardiorespiratory fitness based on sex, age, and VO_2_max. As expected, subjects in the highest fitness tertile had the highest mean M-value (7.0 ± 0.4 mg/kg/min) while those in the middle fitness tertile had the second highest mean M-value (5.9 ± 0.3 mg/kg/min), and those in the lowest tertile had the lowest mean M-value (4.7 ± 0.4 mg/kg/min). Between tertile comparisons of mean M-value showed significant differences between highest and middle fitness tertile groups (p = 0.013), between highest and lowest fitness tertile groups (p < 0.001), and between the middle and lowest fitness tertile groups (p = 0.002).

**Figure 2:**
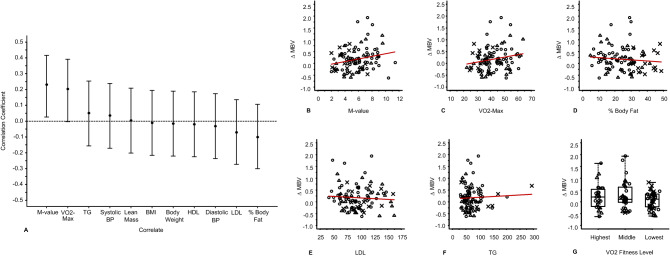
Bivariate relationships between ΔMBV and prediction variables. (**A**) Pearson correlations between ΔMBV and subject characteristics. The vertical lines indicate the 95% confidence interval for the correlation coefficient. (**B**–**F**) Relationships between ΔMBV and individual predictor variables. The red lines represent the ordinary least squares linear regressions. (**G**) Relationship between ΔMBV and cardiorespiratory fitness. BMI, body mass index; BP, blood pressure; HDL, high-density lipoprotein cholesterol; LDL, low density lipoprotein cholesterol; TRI, triglycerides; VO_2_max, maximum consumption of oxygen (mL/kg/min); VO2 fitness, level of cardiorespiratory fitness by tertile. Plotting symbols: Healthy°, Obese˟, Type 1 diabetes^Δ^.

Analyzing the association between ΔMBV and phenotypic variables (Fig. 2), only M-value (r = 0.23, 95% CI [0.03, 0.42], p = 0.023) and VO_2_max (r = 0.20, 95% CI [0.00, 0.39], p = 0.047) correlated with ΔMBV. The other variables including measures of body weight and body composition, LDL-C, triglyceride, and baseline insulin levels, which correlated with M-value, did not correlate with ΔMBV. There was no difference among the fitness tertiles in terms of ΔMBV (p = 0.23, Fig. 2G). There was also no significant correlation between fasting plasma insulin levels and ΔMBV in the subjects without type 1 diabetes (r = − 0.18; 95% CI [− 0.4, 0.05], p = 0.12).

The multivariate regression model type III ANOVA F-tests for testing the null hypothesis is presented in Table 2. With respect to M-value, the metabolic insulin resistance risk factors as a unit provided significant predictive value. Both plasma triglyceride levels and ΔMBV were unique predictors of M-value. With respect to ΔMBV, the metabolic insulin resistance risk factors as a unit did not provide significant predictive information. Only M-value independently predicted ΔMBV. Despite the positive association between VO_2_max and M-values and the positive association between VO_2_max and ΔMBV, cardiorespiratory fitness tertile did not provide significant predictive information about M-value or ΔMBV.

**Table 2:** Multivariate model ANOVA summary.

Predictor	Degrees of freedom	Partial sum of squares	Mean square error	Type III F-statistics	P-value
**Bivariate relationships between predictor variables and M-value**					
BMI	1	0.87	0.87	0.31	0.581
Body weight	1	5.63	5.63	2.00	0.161
VO_2_ fitness	2	5.80	2.90	1.03	0.361
Lean body mass	1	0.63	0.63	0.23	0.636
% body fat	1	4.85	4.85	1.72	0.193
Systolic BP	1	6.31	6.31	2.24	0.138
Diastolic BP	1	1.65	1.65	0.59	0.446
LDL	1	0.04	0.04	0.01	0.907
HDL	1	6.08	6.08	2.16	0.145
Triglycerides	1	13.95	13.95	4.95	0.029
Diabetes	1	5.24	5.24	1.86	0.176
ΔMBV	1	11.67	11.67	4.14	0.045
Total	13	174.80	13.45	4.78	< 0.001
Error	83	233.70	2.82		
**Bivariate relationships between predictor variables and insulin-mediated change in skeletal muscle MBV (ΔMBV)**					
BMI	1	0.08	0.08	0.34	0.563
Body weight	1	0.18	0.18	0.73	0.394
VO_2_ fitness	2	0.66	0.33	1.33	0.270
Lean body mass	1	0.49	0.49	2.00	0.161
% body fat	1	0.25	0.25	1.00	0.319
Systolic BP	1	0.04	0.04	0.16	0.695
Diastolic BP	1	0.00	0.00	0.00	0.988
LDL	1	0.08	0.08	0.32	0.571
HDL	1	0.02	0.02	0.09	0.767
Triglycerides	1	0.31	0.31	1.26	0.264
M-value	1	1.02	1.02	4.14	0.045
Diabetes	1	0.00	0.00	0.01	0.943
Total	13	2.90	0.22	0.90	0.553
Error	83	20.50	0.25		

BMI, body mass index; VO_2_ fitness, tertile of cardiorespiratory fitness with 1 representing the highest and 3 representing the lowest level of fitness; LDL, low-density lipoprotein cholesterol; HDL, high-density lipoprotein cholesterol.

**Figure 3:**
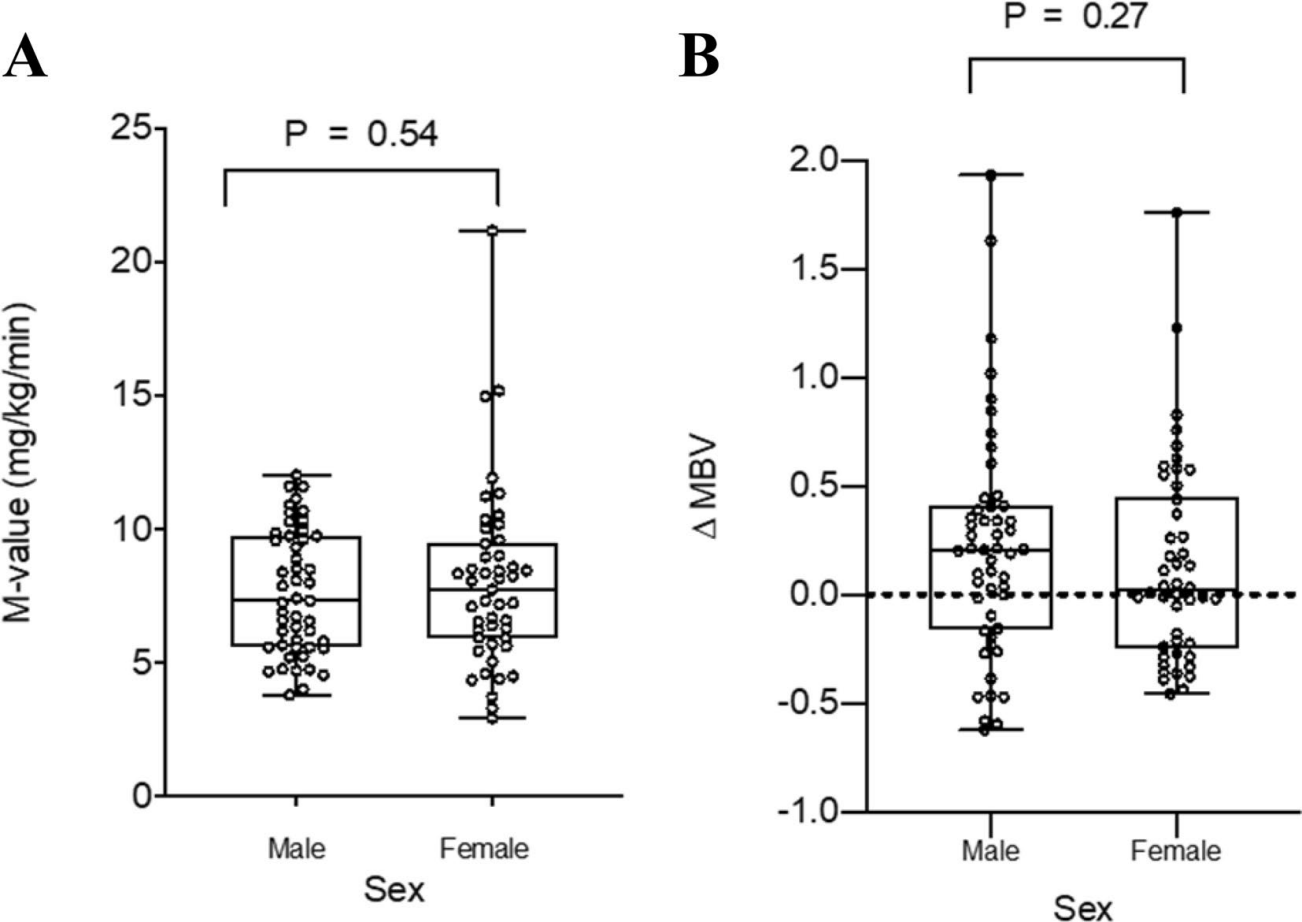
Sex comparisons between M-values corrected for lean body mass (**A**) and ΔMBV (**B**). P-values represent unpaired t-tests with Welch’s correction.

M-values and ΔMBV were compared between males and females to assess for any sex difference. As expected, our study participants had a large range of insulin sensitivity patterns. Thus, the glucose infusion rates during insulin clamp spanned over a large range for both men and women and the ∆MBV responses ranged from marked increase, no change to even a decrease (Fig. 3). Based on total body weight, females had a lower M-value compared with men (5.25 ± 0.28 vs. 6.21 ± 0.29 mg/kg/min, p = 0.038), but this difference disappeared after lean body mass correction (Fig. 3A). There was clearly no difference in mean ΔMBV between males and females (0.22 ± 0.07 v. 0.13 ± 0.07 respectively, p = 0.271, Fig. 3B).

## Discussion

While we and others have previously shown a close interplay between muscle microvascular and metabolic insulin action and resistance in both humans and laboratory rodents, there has been no study of phenotypic predictors of microvascular insulin responses. Using multivariate model the current study for the first time showed that microvascular and metabolic insulin action independently predict one another in humans with a range of insulin sensitivity. To avoid confounding from many other conditions associated with insulin resistance, we limited our study population to young adults and included only those who are healthy and lean, those with class 1 obesity who are otherwise healthy and without family history of diabetes, and those with controlled T1D, a population exhibiting insulin resistance although typically without significant metabolic disarrays. While normoglycemic participants with obesity had the greatest degree of metabolic insulin resistance and microvascular insulin resistance compared to the other two groups with essentially no increase in MBV following insulin, there is a clear heterogeneity in microvascular insulin responses in our study population. Most but not all lean healthy people exhibit an insulin-mediated increase in microvascular perfusion and on the contrary many obese and a few lean healthy and T1D subjects even displayed a decrease in MBV after insulin infusion, consistent with insulin-mediated vasoconstriction. This heterogeneity argues strongly for an urgent need to identify phenotypic predictors of microvascular insulin responses in humans.

That insulin-mediated changes in muscle microvascular blood volume independently predict insulin stimulated whole body glucose disposal (primarily in the skeletal muscle) is consistent with prior data demonstrating that abolishing the insulin-mediated increase in leg blood flow and muscle microvascular perfusion by inhibiting nitric oxide synthase reduces leg glucose uptake by ~ 33% in humans^29^ and 40% in rodents^14^. In a preclinical study of male Sprague–Dawley rats, high-fat diet impaired insulin-mediated microvascular blood flow, measured by CEU, in as little as 3 days whereas diminished whole-body glucose utilization was not detectable until 7 days^16^. Conversely, preventing or reversing microvascular insulin resistance using a variety of approaches, including muscle contraction^30^, adiponectin^19^, GLP-1 or GLP-1 receptor agonist^31,32^, angiotensin II receptor antagonist^33^, and inhibition of vascular inflammation^16^, all enhanced metabolic insulin responses in insulin resistant rodents. These data suggest strongly that insulin’s microvascular action closely couples with its metabolic action, and microvascular insulin resistance precedes metabolic insulin resistance during the development of systemic insulin resistance. Thus, microvascular insulin resistance is a potential therapeutic target for diabetes prevention, management, and averting cardiovascular complications.

We are intrigued to find that M-value provided unique predictive information about ΔMBV. This is not surprising given that almost all commonly recognized causes of metabolic insulin resistance associate with or induce muscle microvascular insulin resistance^2,16,17^ and insulin induces both vasodilatory microvascular recruitment and muscle glucose uptake via the common insulin receptor/IRS/PI3-kinase signaling pathway^13,34^. Therefore, resistance-inducing factors can act on the insulin signaling on both myocytes and endothelial cells to induce insulin resistance.

In our study population, VO_2_max correlated with both M-value and ΔMBV but cardiorespiratory fitness did not provide unique predictive information about either metabolic or microvascular insulin sensitivity when we included other conventional insulin resistance predictor variables in the regression model. This is likely due to the fact that cardiorespiratory fitness is the result of complex physiologic interplays among many factors, including physical activity. Indeed, exercise provides a myriad of salutary cardiometabolic effects which result in improved all-cause and cardiovascular mortality in diabetes^35^. Just one exercise session prevented FFA-induced insulin resistance in healthy young women^36^. While the beneficial effects of acute exercise on insulin sensitivity are apparent, particularly in subjects with obesity/sedentary lifestyle, prospective data regarding the relationship between cardiorespiratory fitness (VO_2_max or peak oxygen consumption, VO_2_peak) and metabolic insulin sensitivity is nevertheless somewhat mixed. In adolescents without diabetes (n = 122) cardiorespiratory fitness correlated positively with glucose disposal rate but this relationship disappeared after adjusting for differences in adiposity^37^. Similarly, reduced waist circumference, but not improved cardiorespiratory fitness, predicted insulin sensitivity in participants (n = 59) with abdominal obesity assigned to exercise training^38^. In the recent GO-ACTIWE study, participants (n = 100) with a BMI in the overweight/class I obesity range were randomized to control or three different exercise protocols of varying intensity^39^. All three exercise groups showed an improvement in peripheral insulin sensitivity (M-value divided by steady state plasma insulin level), cardiorespiratory fitness, and abdominal adiposity at the end of 6 months^39^. Conversely, in one small prospective study (n = 26) which included men who were lean, obese, or had diet-controlled T2D, exercise training improved cardiorespiratory fitness but not M-value^40^. Further studies are needed to accurately define the interplays between cardiorespiratory fitness and the muscle insulin action.

The relationship between ΔMBV and VO_2_max has not been well defined, but the positive association between ΔMBV and VO_2_max observed in the current study is consistent with a prior report of such a correlation in a small subset (n = 8) of healthy humans receiving lipid infusion^17^. It appears that both acute and chronic exercise impact muscle microvascular perfusion. Studies in both rodents^30^ and healthy humans^41^ showed that even low intensity muscle contraction can potently increase skeletal muscle microvascular perfusion, while exercise training improves skeletal muscle capillary density and microvascular blood flow after a glucose load in people with T2D^42^. When we divided all subjects into 3 tertiles based on the cardiorespiratory fitness levels we saw that higher fitness level corresponded to higher mean M-value and lower fitness level with lower mean M-value. Interestingly this “dose response” pattern was absent between cardiorespiratory fitness and ΔMBV. This may relate to higher baseline microvascular perfusion in individuals at the highest cardiorespiratory fitness level so that insulin may not have increased microvascular blood volume to the same degree in highly fit compared to moderately fit subjects. However, baseline forearm skeletal muscle MBV was not significantly different across fitness groups (highest 5.0 ± 0.7, middle 3.4 ± 0.4, and lowest 4.3 ± 0.4 VI units; p = 0.1). Another possible explanation is that for people with high cardiorespiratory fitness there is no benefit to further increase microvascular perfusion while people in the lowest tertile have microvascular insulin resistance, as evidenced by lower insulin-mediated glucose disposal in these subjects, and less increase in ΔMBV. Finally, it is certainly possible that participants predominately exercised an alternative muscle group (i.e. gastrocnemius or quadriceps muscles) which could affect the cardiorespiratory fitness level but was not assessed by our study of forearm muscle perfusion.

Fasting insulin levels correlated strongly with M-value but were not included in the multivariate analysis due to the fact we did not assay for insulin analog concentrations in subjects with T1D. Our finding is consistent with multiple prior studies demonstrating that fasting insulin levels strongly predict M-value in participants with normoglycemia, impaired glucose tolerance, and non-insulin dependent diabetes^7,43^. The lack of correlation between fasting plasma insulin levels and ΔMBV in the subjects without T1D is not surprising as fasting plasma insulin levels predominantly reflect hepatic insulin sensitivity and β-cell function^6^.

In the current study we did not see a sex difference for insulin-mediated changes in muscle microvascular perfusion, i.e., ΔMBV. Similarly the M-value corrected for lean body weight did not differ between women and men. Here prior data is mixed with the CACTI cohort study showing woman without diabetes had a higher M-value compared to men^44^ and this finding was also previously seen in young adults after correction for lean body mass^45^ but others saw no difference^46^. In a subgroup analysis of the lean/healthy participants, we found no sex difference in M-value corrected for lean body mass (males 8.2 ± 0.4 v. females 7.5 ± 0.5 mg/kg/min; p = 0.3).

In the current study eight individuals with T1D required a low-dose insulin infusion, starting two hours prior to vascular and clamp studies, to bring glucose into target level, which could potentially alter subsequent insulin-mediated GIR and MBV. We believe this is less likely as only 8 out of 25 subjects required low dose insulin infusion, the insulin dose was only 1/10th of the dose used during the insulin clamp, and the T1D group had an average GIR that was only slightly lower than the lean group. Additionally participants with T1D did not appear to have higher baseline MBV compared to lean and obese subjects, suggesting that low dose insulin infusion did not increase baseline MBV.

Our study has several limitations. We did not include fasting insulin level as a predictor variable in the multivariate analysis due to the fact that all subjects with T1D were on insulin analogues and the basal plasma levels were not determined. Further, addition of C-reactive protein (CRP) could shed more light on the role of inflammation in microvascular insulin responses given that CRP independently associates with metabolic insulin resistance^47^ and predicts cardiovascular disease in humans^48^. Additionally, we did not include several other indices such as free fatty acids and branched-chain amino acid byproducts, the former which induces metabolic and microvascular insulin resistance in healthy humans^28^ and the latter which compellingly correlates with metabolic insulin resistance in populations with class I obesity^49^. These are all important to consider for future studies. Also, acute exercise increases insulin-mediated glucose disposal after 24 to 48 h in some^50^ but not all studies^51,52^, and this may have led to higher M-values in participants who exercised within this timeframe. Timing of most recent exercise was not recorded in this study but is an important consideration for future studies. Additionally, poor ethnic diversity represents a significant limitation in this study. Although much remains to be elucidated in populations with comorbidities like diabetes and obesity, healthy individuals who are black have reduced medium-size vessel and microvascular endothelial function compared to white individuals also studied in the United States^53,54^. Finally, we limited our study to a relatively young population to avoid confounding influence of other comorbidities frequently seen in older humans such as hypertension, hyperlipidemia, and metabolic syndrome. Whether our study findings pertain to a more ethnically diverse population, older population, or to persons with other co-morbidities remains to be defined. On the other hand, including individuals with T1D, a population with under-recognized insulin resistance, is a major strength of this study particularly in light of the knowledge gaps in pathophysiology of insulin resistance and cardiovascular disease in T1D.

## Conclusions

Metabolic and microvascular insulin responses are important mutual predictors in humans, at least in populations that include people who are lean, have class 1 obesity or controlled T1D but without other major comorbidities. However, most phenotypic predictors of metabolic insulin resistance do not predict microvascular insulin responsiveness. More prospective research is needed to understand the underlying mechanisms regulating microvascular insulin action and resistance.

## Data Availability

The datasets used and/or analyzed in the current study are available from the corresponding author upon reasonable request.

## Abbreviations

BF: Percent body fat
BMI: Body mass index
CEU: Contrast enhanced ultrasound
INS: Insulin
LDL-C: LDL cholesterol
MBV: Microvascular blood volume
T1D: Type 1 diabetes
T2D: Type 2 diabetes
TG: Triglycerides
VO_2_max: Maximum consumption of oxygen
WT: Total body weight

## References

[1] Kaul K, Apostolopoulou M, Roden M (2015). Insulin resistance in type 1 diabetes mellitus. Metabolism.

[2] Clerk LH (2006). Obesity blunts insulin-mediated microvascular recruitment in human forearm muscle. Diabetes.

[3] Gregory JM, Cherrington AD, Moore DJ (2020). The peripheral peril: Injected insulin induces insulin insensitivity in type 1 diabetes. Diabetes.

[4] Bressler P, Bailey SR, Matsuda M, DeFronzo RA (1996). Insulin resistance and coronary artery disease. Diabetologia.

[5] Bjornstad P (2016). Estimated insulin sensitivity predicts incident micro- and macrovascular complications in adults with type 1 diabetes over 6 years: The coronary artery calcification in type 1 diabetes study. J. Diabetes Complicat..

[6] Matthews DR (1985). Homeostasis model assessment: Insulin resistance and beta-cell function from fasting plasma glucose and insulin concentrations in man. Diabetologia.

[7] McAuley KA (2001). Diagnosing insulin resistance in the general population. Diabetes Care.

[8] Katz A (2000). Quantitative insulin sensitivity check index: A simple, accurate method for assessing insulin sensitivity in humans. J. Clin. Endocrinol. Metab..

[9] Caro JF (1987). Insulin receptor kinase in human skeletal muscle from obese subjects with and without noninsulin dependent diabetes. J. Clin. Investig..

[10] Duncan JG, Fong JL, Medeiros DM, Finck BN, Kelly DP (2007). Insulin-resistant heart exhibits a mitochondrial biogenic response driven by the peroxisome proliferator-activated receptor-alpha/PGC-1alpha gene regulatory pathway. Circulation.

[11] Groop LC (1989). Glucose and free fatty acid metabolism in non-insulin-dependent diabetes mellitus. Evidence for multiple sites of insulin resistance. J. Clin. Investig..

[12] Basu R, Chandramouli V, Dicke B, Landau B, Rizza R (2005). Obesity and type 2 diabetes impair insulin-induced suppression of glycogenolysis as well as gluconeogenesis. Diabetes.

[13] Barrett EJ (2009). The vascular actions of insulin control its delivery to muscle and regulate the rate-limiting step in skeletal muscle insulin action. Diabetologia.

[14] Vincent MA (2004). Microvascular recruitment is an early insulin effect that regulates skeletal muscle glucose uptake in vivo. Diabetes.

[15] Baron AD, Laakso M, Brechtel G, Edelman SV (1991). Mechanism of insulin resistance in insulin-dependent diabetes mellitus: A major role for reduced skeletal muscle blood flow. J. Clin. Endocrinol. Metab..

[16] Zhao L (2015). Inflammation-induced microvascular insulin resistance is an early event in diet-induced obesity. Clin. Sci..

[17] Eggleston EM, Jahn LA, Barrett EJ (2013). Early microvascular recruitment modulates subsequent insulin-mediated skeletal muscle glucose metabolism during lipid infusion. Diabetes Care.

[18] Chan A, Barrett EJ, Anderson SM, Kovatchev BP, Breton MD (2012). Muscle microvascular recruitment predicts insulin sensitivity in middle-aged patients with type 1 diabetes mellitus. Diabetologia.

[19] Zhao L (2015). Globular adiponectin ameliorates metabolic insulin resistance via AMPK-mediated restoration of microvascular insulin responses. J. Physiol..

[20] Chai W (2011). Salsalate attenuates free fatty acid-induced microvascular and metabolic insulin resistance in humans. Diabetes Care.

[21] Subaran SC (2014). GLP-1 at physiological concentrations recruits skeletal and cardiac muscle microvasculature in healthy humans. Clin. Sci..

[22] Wang N (2019). Vasodilatory actions of glucagon-like peptide 1 are preserved in skeletal and cardiac muscle microvasculature but not in conduit artery in obese humans with vascular insulin resistance. Diabetes Care.

[23] Pescatello, L., Arena, R., Riebe, D. & Thompson, P. Vol. III, 88–92 (Lippincott Williams & Wilkins, 2014).

[24] James D, Umekwe N, Edeoga C, Nyenwe E, Dagogo-Jack S (2020). Multi-year reproducibility of hyperinsulinemic euglycemic clamp-derived insulin sensitivity in free-living adults: Association with incident prediabetes in the POP-ABC study. Metabolism.

[25] Ferrannini E (1985). Effect of insulin on the distribution and disposition of glucose in man. J. Clin. Investig..

[26] Tan AWK (2018). GLP-1 and insulin recruit muscle microvasculature and dilate conduit artery individually but not additively in healthy humans. J. Endocr. Soc..

[27] Keske MA, Clerk LH, Price WJ, Jahn LA, Barrett EJ (2009). Obesity blunts microvascular recruitment in human forearm muscle after a mixed meal. Diabetes Care.

[28] Liu Z, Liu J, Jahn LA, Fowler DE, Barrett EJ (2009). Infusing lipid raises plasma free fatty acids and induces insulin resistance in muscle microvasculature. J. Clin. Endocrinol. Metab..

[29] Sjøberg KA (2017). Exercise increases human skeletal muscle insulin sensitivity via coordinated increases in microvascular perfusion and molecular signaling. Diabetes.

[30] Inyard AC, Chong DG, Klibanov AL, Barrett EJ (2009). Muscle contraction, but not insulin, increases microvascular blood volume in the presence of free fatty acid-induced insulin resistance. Diabetes.

[31] Chai W, Zhang X, Barrett EJ, Liu Z (2014). Glucagon-like peptide 1 recruits muscle microvasculature and improves insulin's metabolic action in the presence of insulin resistance. Diabetes.

[32] Chai W, Fu Z, Aylor KW, Barrett EJ, Liu Z (2016). Liraglutide prevents microvascular insulin resistance and preserves muscle capillary density in high-fat diet-fed rats. Am. J. Physiol. Endocrinol. Metab..

[33] Chai W (2010). Angiotensin II type 1 and type 2 receptors regulate basal skeletal muscle microvascular volume and glucose use. Hypertension.

[34] Cheatham B (1994). Phosphatidylinositol 3-kinase activation is required for insulin stimulation of pp70 S6 kinase, DNA synthesis, and glucose transporter translocation. Mol. Cell Biol..

[35] Kodama S (2013). Association between physical activity and risk of all-cause mortality and cardiovascular disease in patients with diabetes: A meta-analysis. Diabetes Care.

[36] Schenk S, Horowitz JF (2007). Acute exercise increases triglyceride synthesis in skeletal muscle and prevents fatty acid-induced insulin resistance. J. Clin. Investig..

[37] Lee S, Bacha F, Gungor N, Arslanian SA (2006). Cardiorespiratory fitness in youth: Relationship to insulin sensitivity and beta-cell function. Obesity.

[38] Brennan AM, Lam M, Stotz P, Hudson R, Ross R (2014). Exercise-induced improvement in insulin sensitivity is not mediated by change in cardiorespiratory fitness. Diabetes Care.

[39] Blond MB (2019). How does 6 months of active bike commuting or leisure-time exercise affect insulin sensitivity, cardiorespiratory fitness and intra-abdominal fat? A randomised controlled trial in individuals with overweight and obesity. Br. J. Sports Med..

[40] Segal KR (1991). Effect of exercise training on insulin sensitivity and glucose metabolism in lean, obese, and diabetic men. J. Appl. Physiol..

[41] Vincent MA (2006). Mixed meal and light exercise each recruit muscle capillaries in healthy humans. Am. J. Physiol. Endocrinol. Metab..

[42] Russell RD (2017). Skeletal muscle microvascular-linked improvements in glycemic control from resistance training in individuals with type 2 diabetes. Diabetes Care.

[43] Laakso M (1993). How good a marker is insulin level for insulin resistance?. Am. J. Epidemiol..

[44] Millstein RJ (2018). Sex-specific differences in insulin resistance in type 1 diabetes: The CACTI cohort. J. Diabetes Complicat..

[45] Dengel DR, Jacobs DR, Steinberger J, Moran AM, Sinaiko AR (2011). Gender differences in vascular function and insulin sensitivity in young adults. Clin. Sci..

[46] Goodpaster BH, Thaete FL, Simoneau JA, Kelley DE (1997). Subcutaneous abdominal fat and thigh muscle composition predict insulin sensitivity independently of visceral fat. Diabetes.

[47] Festa A (2000). Chronic subclinical inflammation as part of the insulin resistance syndrome: The Insulin Resistance Atherosclerosis Study (IRAS). Circulation.

[48] Haverkate F, Thompson SG, Pyke SD, Gallimore JR, Pepys MB (1997). Production of C-reactive protein and risk of coronary events in stable and unstable angina. European Concerted Action on Thrombosis and Disabilities Angina Pectoris Study Group. Lancet.

[49] Newgard CB (2009). A branched-chain amino acid-related metabolic signature that differentiates obese and lean humans and contributes to insulin resistance. Cell Metab..

[50] Perseghin G (1996). Increased glucose transport-phosphorylation and muscle glycogen synthesis after exercise training in insulin-resistant subjects. N. Engl. J. Med..

[51] Wadley GD (2007). Increased insulin-stimulated Akt pSer473 and cytosolic SHP2 protein abundance in human skeletal muscle following acute exercise and short-term training. J. Appl. Physiol..

[52] Cusi K (2000). Insulin resistance differentially affects the PI 3-kinase- and MAP kinase-mediated signaling in human muscle. J. Clin. Investig..

[53] Patik JC (2018). Sex differences in the mechanisms mediating blunted cutaneous microvascular function in young black men and women. Am. J. Physiol. Heart Circ. Physiol..

[54] Ozkor MA (2014). Differences in vascular nitric oxide and endothelium-derived hyperpolarizing factor bioavailability in blacks and whites. Arterioscler. Thromb. Vasc. Biol..

